# Clinical Observations of the Effectiveness of the Masquelet Induced Membrane Technique in the Treatment of Critical Long-Bone Defects of the Lower and Upper Extremities

**DOI:** 10.3390/medicina60121933

**Published:** 2024-11-24

**Authors:** Kamil Kołodziejczyk, Aleksander Ropielewski, Rafał Garlewicz, Marcin Złotorowicz, Jarosław Czubak

**Affiliations:** Department of Replantation and Reconstruction, Centre of Postgraduate Medical Education, Professor A. Gruca Teaching Hospital, Konarskiego 13, 05-400 Otwock, Poland

**Keywords:** Masquelet technique, induced membrane, bone reconstruction, lower extremity, bone infection, bone non-union

## Abstract

*Background and Objectives:* Successful treatment of severe trauma and fractures of the long bones with successful healing and bone union is still a significant challenge for surgeons. Unfortunately, up to 10% of long-bone fractures develop bone healing disorders. The aim of this study was to evaluate the results of treating bone defects with different etiologies in the upper and lower extremities using the induced membrane technique. *Materials and Methods:* We prospectively evaluated the radiological and clinical outcomes of 45 patients with severe bone defects treated with the induced membrane technique during the period from May 2021 to October 2023. The time to bone defect regeneration, size of the bone defect, and the cost of treatment were evaluated. Functional outcomes were assessed using the Disabilities of the Arm Shoulder and Hand (DASH) scale, SF-36, and the Lower Limb Functional Index (LLFI). *Results:* The mean follow-up time was 31 months (12–35). There were 20 patients with upper extremity bone defects and 25 with lower extremity bone defects. The mean defect length was 7.9 cm for the upper extremity (3.5–18) and 5.3 cm for the lower extremity (3–11). The mean times to achieve bone union and remodeling were 6.0 months (3–12) and 9 months (3–13) for the upper and lower limbs, respectively. Clinical evaluation at the end of treatment (achieving bone union) showed statistically significant improvements in the DASH, SF-36, and LLFI scales for pre- and postoperative outcomes. There was no statistical significance in the SF-36 clinical scale scores after surgical treatment compared to reconstructive treatment of upper and lower extremity bone defects. *Results:* The presented reconstructive approach to the treatment of bone defects and healing disorders and extensive analysis demonstrate the effectiveness of the induced membrane technique in a short follow-up period, with a relatively high level of patient comfort and good clinical results in the treatment of severe bone defects with particularly infectious etiologies.

## 1. Introduction

The development of bone union failure regularly results in prolonged pain and limited function of the affected extremity, requiring a varied treatment strategy. Due to the many clinical situations and unique patient-dependent factors, there is no universal treatment method for critical bone defects (these are usually larger than 3 cm and regarded as unlikely to heal spontaneously despite surgical stabilization, requiring further surgical intervention such as autologous bone grafting or reconstructive techniques) [1,2]. Modern bone fixation methods, an understanding of bone biology, and advances in surgical techniques and infection control offer a chance to help patients with critical bone defects and bone non-union, which in the past would often have required amputation. There are several options such as the induced membrane technique (IMT) described by A. Masquelet, which consists of two stages of treatment [3]. In the first stage, radical debridement and segmental resection of devitalized bone of the pseudarthrosis joint is performed; the defect is replaced with polymethylmethacrylate (PMMA) bone cement with an antibiotic. In the second stage, the PMMA is replaced with bone grafts. Other methods for treating bone defects and pseudarthrosis joints include bone transfer and vascularized or non-vascularized bone grafts using internal or external fixation methods [4,5,6,7]. The magnitude of the non-union problem is difficult to determine precisely, but it is estimated that 5–10% of fractures evolve to non-union, and a much smaller percentage requires bone reconstruction techniques [4,5,6]. Long-bone non-union complicated by critical long-bone defect occurs in about 3% of fractures [2]. Nicotinism, diabetes, old age, osteoporosis, high-energy trauma, vascularization, and nutritional disorders are considered the main factors for bone non-union. The treatment of non-union fractures is associated with high direct and indirect costs [6,7].

Although there are many studies on IMT and its effectiveness in treating bone defects, there are relatively few prospective studies that also evaluate functional outcomes. The aim of this study is to present a treatment process using a two-stage technique with an induced membrane (Masquelet technique) for patients with bone defects and infected bone pseudarthrosis joints. The research hypothesis is to evaluate the impact and utility of the induced membrane on achieving bone union in long-term pseudarthrosis joints of long bones.

## 2. Materials and Methods

The study was approved by the Institutional Ethics Committee. Informed consent was obtained from all patients participating in the study.

In this prospective study, the efficacy of IMT in treatment of bone defects was evaluated. A total of 45 patients with bone loss in the upper (*n* = 20; 44%) and lower (*n* = 25; 56%) extremities, operated on between May 2021 and October 2023, were included in the study.

Patient data collected included the bone affected, age, gender, smoking status, comorbidities that may cause non-union (diabetes mellitus, rheumatoid arthritis), osteitis and the bacteria causing it, defect size, interval and type of fixation between first-stage (S1) and second-stage (S2) IMT treatments, and time to bone consolidation.

We analyzed the effectiveness and time to bone graft consolidation, the bone formation index (a value that is the ratio of the bone graft consolidation time to the length of the bone defect; the index indicates the expected time for bone graft consolidation dependent on the location and length of the defect), observed complications, surgery time, membrane induction, hospitalization costs, and bacteriological data. Functional outcomes were assessed using the SF-36, LLFI, and DASH questionnaire [8,9,10]. Functional outcomes were measured before the first stage of surgery and after achieving bone fusion and the extremity returning to full weight bearing. Bone union was defined as the consolidation of the regenerated bone in two X-ray projections and the possibility of pain-free weight bearing on the operated extremity. All patients were administered the SF-36 questionnaire and the DASH scale for the upper limb and LLFI for the lower extremity.

The inclusion criteria included a critical bone defect (>3 cm) and defects resulting from trauma or septic treatment or aseptic non-union or pseudarthrosis of long bone. Exclusion criteria included loss of follow-up and oncological etiology.

### 2.1. Statistical Analysis

In accordance with the assumption of maintaining a 95% confidence interval, a test power of 80%, and an error of 5% with an adult population of about 37 million, a study group size of about 45 patients was estimated. Based on the Shapiro–Wilk test, the variables were not normally distributed; therefore, we used the Wilcoxon signed-rank test. The analysis of the data included descriptive statistics and was performed in Stata v. 11.0 (StataCorp, College Station, TX, USA) and Excel (Microsoft, Redmond, Washington, DC, USA). The significance level was set at *p* < 0.05. 

### 2.2. Surgical Technique

**Figure 1:**
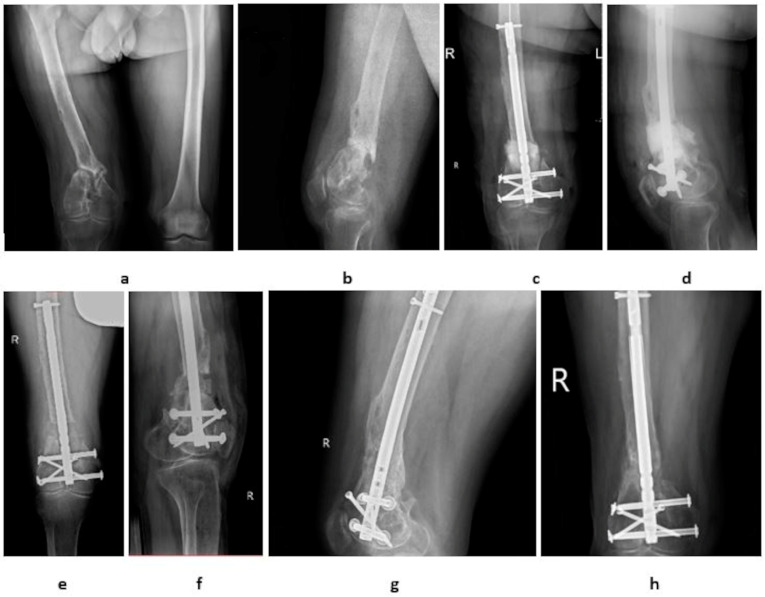
A 37-year-old man with post-traumatic pseudoarthritis of the right femur (4 years after injury). (**a**,**b**) preoperative femoral pseudoarthritis; (**c**,**d**) first-stage IMT postoperative X-ray, resection of pseudoarthritis, IMN and PMMA (with clindamycin, gentamycin, vancomycin); (**e**,**f**) second-stage IMT postoperative X-ray, removal of PMMA, autograft of iliac crest bone grafting; (**g**,**h**) healthy bore union of new distal femur.

**Figure 2:**
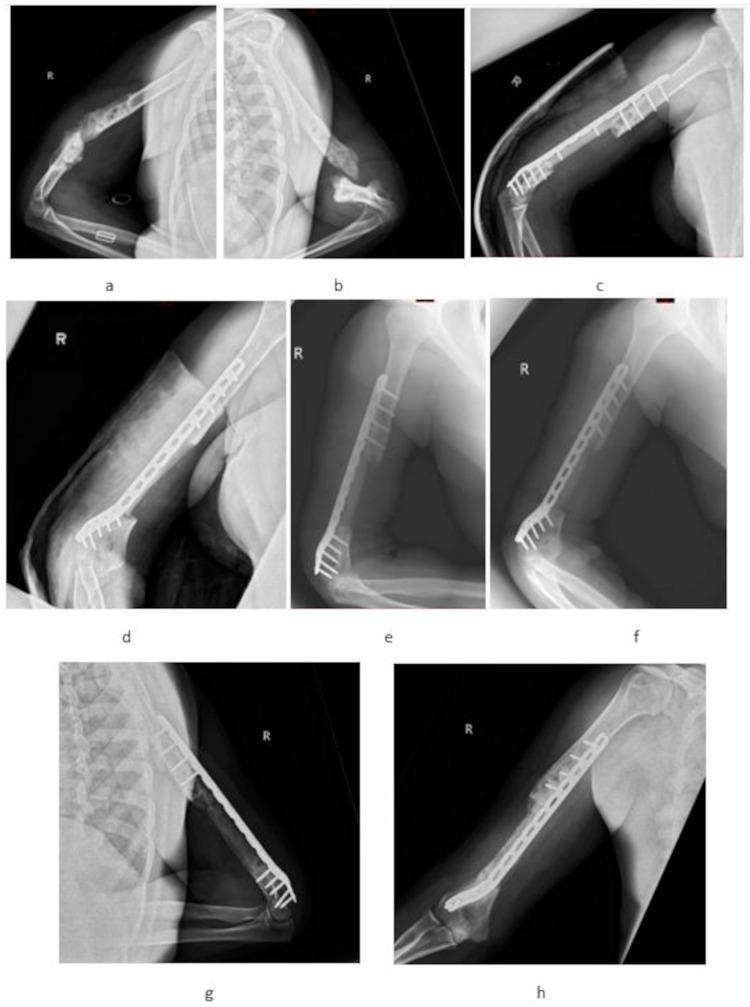
A 48-year-old female with post-traumatic pseudoarthritis of the right humerus (5 years after injury). (**a**,**b**) preoperative X-rays; (**c**,**d**) first-stage IMT postoperative X-ray humerus stabilization with long titanium LCP with PMMA (with clindamycin, gentamycin, vancomycin); (**e**,**f**) second-stage IMT postoperative X-ray autologous femoral RIA graft input to vascularized induced membrane; (**g**,**h**) healthy humerus bone and radiological union.

All patients were treated according to the modified Masquelet technique. Unlike the original method, PMMA with gentamicin, clindamycin, and vancomycin was used and internal bone fixation methods were applied [3,11]. In the first stage of treatment, radical segmental resection of the bone non-union, including the adjacent bone up to the “paprika sign” (bone material for microbiological examination), was performed. Bone fixation was then performed, and the defect was filled with PMMA with antibiotics. Depending on the location of the bone defect, an intramedullary nail (IMN) or low-contact locking plate (LCP) was used as fixation, as shown in Figure 1 and Figure 2, respectively. The second stage was performed after approximately 8–11 weeks. CRP (C-reactive protein) levels were monitored before the second stage. The current bone fixation was left in place, only PMMA was removed (a microbiological examination was also performed), and bone defects were supplemented with autologous bone graft RIA (reaming irrigation aspiration) or graft from the iliac crest (maximum graft up to 7 cm). Autogenous bone graft from the iliac crest was used for bone defects up to 7 cm, while larger defects were restored using autogenous grafts by the RIA procedure.

## 3. Results

All patients had previously undergone several surgical treatments of fracture and bone non-union or pseudarthrosis outside our department (at least two operations previously). The average duration of limb dysfunction due to lack of bone fusion was 63 months for the upper limb and 46 months for the lower limb. The patients’ demographics and characteristics are presented in Table 1.

Bone union was achieved in *n* = 17 (85%) patients for upper extremities and *n* = 23 (92%) for lower extremities. The overall treatment success rate was 88% (bone union was achieved in 40 patients) and the mean follow-up time was 31 months (12–35).

The average times to achieve bone graft consolidation were 6 months (3–12) and 9 months (3–13) for the upper and lower limbs, respectively. The time between stages was 10.0 weeks (6.7–17.9) and 7.9 weeks (4.6–11.4) for the upper and lower extremities, respectively. The mean bone formation rate (cm/month) was 1.06 for upper extremities and 1.75 for lower extremities. In two lower extremity cases (8%), additional procedures were needed: one propeller flap and one gastrocnemius flap. Revision surgery was needed for six patients (30%) who underwent upper extremity surgery—three due to implant failure and two with bone non-union. In the lower extremity, revision surgery was necessary in five patients (20%), with the main reason being recurrence of infection and bone non-union. There were significantly more complications observed in upper than in lower extremities (*p* = 0.026). No other complications were recorded within the observation period. Bone defects were grafted with autologous grafts, namely, RIA, in 8 (40%) and 10 (40%) patients for the upper and lower extremities, respectively. In the other cases, an iliac crest graft was used (i.e., upper extremity, 12 patients (60%); lower extremity, 15 (60%)). All upper extremity cases were fixed with an LCP. For the lower extremity it was a plate in 12 (48%) cases and intramedullary nail in 13 (52%). The operative time was longer for the lower extremities for S1 and shorter for S2, without significant differences. The mean time of hospitalization was 13.7 (10–20) and 14.8 days (10–28) for the upper and lower extremities, respectively, during S1. For S2, these values were 4 (2–10) and 6.6 (5–14) days, respectively. After the first stage of reconstruction, extended hospitalization (10–14 days) resulted from waiting for microbiological results (sonication) and the use of intravenous empirical antibiotic therapy. The preoperative CRP values were 8.22 ng/L (1.4–25.7), and 10.1 ng/L (1.5–31.8) for the upper and lower extremities, respectively. All patients proceeding to the second stage had a normal CRP result (<5 ng/L), which we considered unnecessary data for analysis. For S2 hospitalization, a significant difference was observed in the hospitalization length. The costs of S1 were USD 5836.89 and 6640.27 for the upper and lower extremities, respectively; and for S2, USD 2836.84 and 3059.20, respectively. Detailed data are presented in Table 2 and Table 3.

The most common etiology of non-union and bone loss was bacterial infection, seen in 17 cases (85%) for upper extremities and 19 (76%) for lower extremities. Mixed bacterial flora were cultivated from preoperative and operative specimens, with prevalence of MSSA (methicillin-sensitive Staphylococcus aureus) or MRCNS (methicillin-resistant coagulase-negative Staphylococcus). Enterococci were only observed in lower limb cases, being cultured in 38% of lower extremity bone defects. The microbiological data and antibiotic treatments are presented in Table 4. The total percentage of infected pseudarthrosis joints in our observation was as high as 80% (36 patients).

**Figure 3:**
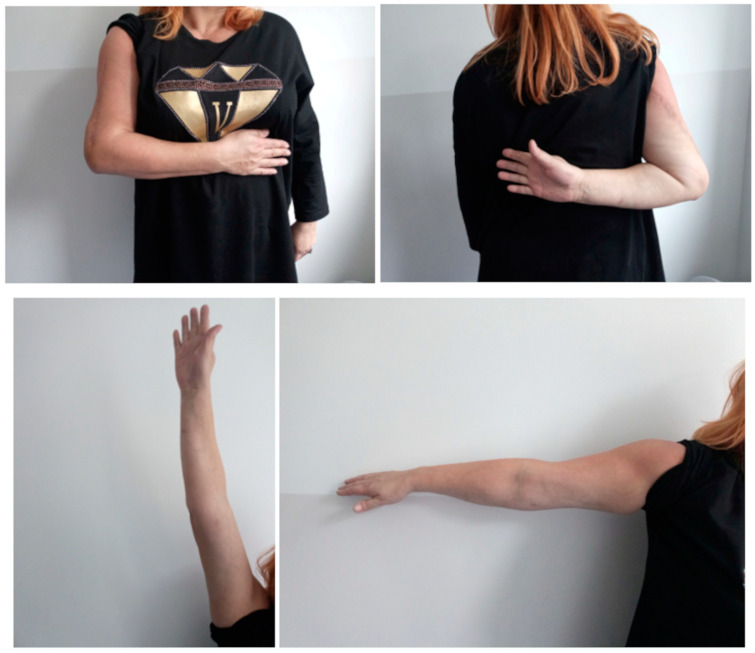
Clinical result of the range of motion of the right upper extremity after surgical treatment of the right humerus bone defect with the the Masquelet technique presented in Figure 2.

Statistically significant improvement in the functional results was observed. An improvement of 56 points was recorded on the DASH scale, and for the LLFI it was 53%. In general life quality metrics, SF-36 physical improved by 30 and 44 points in the upper and lower extremities, respectively, while SF-36 mental improved by 10.5 and 9.85, respectively. All observed changes in the DASH, LLFI, and SF-36 scales were statistically significant. The clinical outcome of the treatment of the right humerus bone defect (Figure 2) is shown in Figure 3. The induced membrane technique allows the length of the limb before the first stage of treatment to be preserved; it does not allow the limb to be lengthened for equalization without increasing the difference in length of the operated limb segment.

There was no statistical difference in the range of improvement in the SF-36 results between the upper and lower extremities. Detailed results are presented in Table 5.

## 4. Discussion

The results show that IMT can be used for treatment of a wide range of clinical conditions with satisfying results. Most of the cases were infected non-unions, and in these situations IMT gives a chance for the infection to heal without risk from external fixation complications such as a higher infection rate and the need for high patient compliance [12,13,14]. When opening the non-union site, one can adequately resect non-vital tissues, which is especially important to prevent bone auto- or allograft lysis due to re-infection. IMT also showed its efficacy in cases of non-infected bone defects or congenital tibia non-union (one patient). The union rate observed in this study is similar or even higher than in previous studies, especially taking into consideration the high prevalence of infectious etiology. Also, the time to bone union and complication rate are comparable with similar papers [15,16,17,18]. Garabano et al. report that polymicrobial infection is associated with a higher risk of re-infection when treating segmental femoral and tibial defects with the Masquelet technique [19]. This article showed that there are no significant differences in time to union or bone union rate depending on autograft source, similarly to comparable studies [20,21]. In all cases in this study, an autologous bone graft was used. For three patients, where the volume of the bone defect was too big, an allogenic bone graft was also used. There are no known studies showing significant difference between autogenic bone grafts compared to autogenic bone mixed with other bone substitutes on union rate [18]; the group size in this study does not allow for significant conclusions to be drawn in this regard. Bone defect length had no effect on the final union in the analyzed group, either in the lower or upper extremities, which is consistent with previous studies [13,22]. However, this phenomenon is observed for the tibia, and one needs to be aware of it when dealing with critical bone defects [23]. Of note is that the bone defects of the femur treated in this study were shorter than in other papers, which may have influenced the study’s results [24,25]. The three patients in whom bone non-union was observed were patients with an infectious etiology of non-union, confirming the importance of remission of infection before the S2 procedure. This was initially highlighted by A. Masquelet and supported by microbiological studies [3,19,20,21,26]. For the same reason, only patients with normal-range CRP levels were qualified for S2 of the IMT.

In difficult cases that do not respond to treatment, or in departments without an experienced surgeon, other treatment methods should be considered, such as bone transport using the Ilizarov technique [27]. Free fibula bone grafting is reserved for specialized centers with microsurgical experience. New treatment methods have also been described, such as osteoinductive autologous bone graft substitutes, which aim to accelerate bone regeneration [28]. The clinical evidence for the use of bone morphogenetic proteins in the treatment of fractures, non-union, necrosis, and bone infection is still controversial, and the number of clinical reports is insufficient [29].

In this study, antibiotic-loaded PMMA was routinely used, which is not advocated by the inventor of the technique, but in his meta-analysis. Lu et al. showed that antibiotic-loaded PMMA spacers shortened time to union [13]. However, the antibiotic concentration can influence membrane formation [30,31]. From a clinical aspect, it is worth mentioning that five (20%) patients were treated for lower extremity bone loss due to the presence of enterococci in surgical material cultures; this needs to be taken into consideration when adjusting empirical treatment. A study by Hofmann et al. showed that gentamicin and vancomycin have no negative effect on osteogenic potential; however, the combination of gentamicin and clindamycin significantly decreased osteogenic potential [32]. This is a very valuable indication that may have further surgical implications. No difference between the type of fixation was observed between an LCP and IMN; however, some studies advocate for the use of IMN fixation whenever possible as it shortens the time to union [33,34]. Although in this study implant failure only occurred in patients fixed with a plate, the surgeon cannot always choose the IMN fixation option. The group size and its heterogeneity do not allow for definitive statements from this study. Similarly to other studies, this work does not suggest that nicotinism or higher age present a higher risk of non-union [35,36,37,38]. A higher rate of implant failure was observed among smokers; however, it is difficult to fully ascribe this to the smoking status as three out of five implant failures were connected with injury or patient non-compliance. The age effect cannot be fully estimated due to the relatively low age of the patients in this study. A very important aspect of this study is the analysis of the functional results. As a prospective study, it was possible to compare pre- and post-treatment results. These show statistically significant differences in SF-36 and the DASH and LLFI scales. No differences are observed between the functional results of SF-36 for the upper and lower extremities. There are few studies on functional results in the literature. Comparing with Grün et al. and extrapolating the results of Raven et al., the functional improvements in this study are comparable [35,39]. We used the LLFI instead of LEFS, as only the LLFI is validated in the Polish language; however, the results are comparable even when taking into consideration the differences between the two scales [9]. Other studies use the Association for the Study and Application of the Method of Illizarov (ASAMI) scale, which is typically used for Illizarov apparatus [40,41]. These significant improvements in the function of the operated extremities does not mean that patients are satisfied with the results, and they often report that the final result does not meet their expectations. This leaves an open question about considering amputation as a method of treatment [42]. However, this matter, although important, is well beyond the scope of this article.

Analyzing the economic cost of treating critical bone defects with IMT, we can only compare the costs with previous studies of this type; this shows that the cost of treatment by IMT is lower than with the Illizarov method, and lower than in comparable studies [43,44,45]. This study has limitations like the heterogeneity typical for IMT patients, and the lack of a comparative group. In addition, the group size does not allow for strong conclusions. The short observation period shows good results, but long-term follow-up could give further insight into the quality of life of IMT-treated patients, functional results, and complications. On the other hand, it is a prospectively planned analysis that takes into consideration many variables with a cost analysis, and it describes functional results as well, which are extremely important in patient treatment and the decision-making process. Our study is a proposal for further observation and open discussion on the reconstructive treatment of bone defects, union disorders, and bone infections using induced membranes.

## 5. Conclusions

This article confirms the versatility and effectiveness of IMT and its predictable results in severe bone defects of various etiologies in both the upper and lower extremities. Although it is a two-stage technique, the use of internal bone fixation makes the whole treatment process relatively comfortable for the patient. The analysis confirms the favorable effect of IMT in the treatment of severe bone defects of especially infectious etiologies with good functional results.

## Figures and Tables

**Table 1:** Demographic data of upper and lower extremity reconstruction using the Masquelet technique.

Variable	Upper Extremity (*n* = 20)	Lower Extremity (*n* = 25)	*p*-Value
Age	44 (15–82)	43 (22–66)	0.73
Sex	Female: 13	Female: 3	0.002
	Male: 7	Male: 22	0.012
Side	Left: 9	Left: 8	0.31
	Right: 11	Right: 17	0.27
Localization	Arm: 7	Femur: 11	----
	Radius: 8	Tibia: 13	----
	Ulna: 5	Fibula: 1	----
Mean disorder history (mth)	63.7 (12–168)	46.7 (3–252)	0.157
Drug and disease	Diabetic: 1 (5%)	Diabetic: 2 (8%)	0.82
	Nicotin: 7 (35%)	Nicotin: 9 (36%)	0.77

mth: month; Wilcoxon signed-rank test, *p*-value < 0.05.

**Table 2:** Analysis of hospitalization and cost data of upper and lower extremity reconstruction using the Masquelet technique.

Variable	Upper Extremity (*n* = 20)	Lower Extremity (*n* = 25)	*p*-Value
Bone fixation	LCP: 20 (100%)	LCP: 12 (48%) IMN: 13 (52%)	---
Bone defect length (mean, cm)	7.95 (3.5–18)	5.28 (3–11)	0.042
Membrane induced	19 (95%)	25 (100%)	0.81
Bone grafting	RIA 8 (40%) Iliac crest 12 (60%)	RIA 10 (40%) Iliac crest 15 (60%)	0.38 0.92
Time to bone union (mean, mth)	6.0 (3–11)	9.1 (3–12)	0.14
Bone union rate (mth/cm)	1068	1.75	0.38
Extra procedures	None	Propeller flap: 1 (4%) Gastrocnemius flap: 1 (4%)	---
CRP before 1st stage (mean, ng/L)	8.22 (1.4–25.7)	10.1 (1.5–31.8)	0.31
Complications	Implant failure: 3 (15%) Non-union: 3 (15%)	Infection return: 3 (12%) Non-union: 2 (8%)	0.026

cm: centimeter; mth: month; LCP: low-contact locking plate; IMN: intramedullary nail; RIA: reaming irrigation aspiration; CRP: C-reactive protein; Wilcoxon signed-rank test, *p*-value < 0.05.

**Table 3:** Analysis of operation data of upper and lower extremity reconstruction using Masquelet technique.

	Upper Extremity (*n* = 20)	Lower Extremity (*n* = 25)	*p*-Value
Operative time—1st stage (mean, min)	178 (90–210)	225 (120–300)	0.35
Hospitalization time—1st stage (mean, day)	13.7 (10–20)	14.8 (10–28)	0.61
Cost—1st stage (mean, $)	5836.89	6640.27	0.70
Operative time—2nd stage (mean, min)	101.5 (60–210)	81.8 (60–110)	0.13
Hospitalization time—2nd stage (mean, day)	4 (2–10)	6.6 (5–14)	0.037
Cost—2nd stage (mean, $)	2836.84	3059.20	0.77
Total cost of 1st and 2nd stages of reconstruction	8673.74	9699.47	0.69

min: minutes; $: USD; Wilcoxon signed-rank test, *p*-value < 0.05.

**Table 4:** Microbiological data and antibiotic treatment.

Variable	Upper Extremity (*n* = 20)	Lower Extremity (*n* = 25)
Aseptic non-union	3 (15%)	6 (24%)
Infected non-union	17 (85%)	19 (76%)
Bacteria	MSSA: 10 MRCNS: 11 MRSA: 2	MSSA: 9 MRCNS: 13 MRSA: 2 *Escherichia coli*: 5 *Escherichia faecalis*: 4
Antibiotic PMMA	gentamycin clindamycin vancomycin	
Antibiotic iv.	Empirical: amikacin, cefuroxim Targeted: vancomycin, cloxacilin	Empirical: amikacin, cefuroxim Targeted: vancomycin, cloxacilin, ciprofloxacin, linezolid
Antibiotic po.	cloxacilin sulfamethoxazolum + trimethoprimum rifampicin	

PMMA: polymethylmethacrylate cement; iv.: intravenous; po.: per oss; MSSA: methicillin-sensitive *Staphylococcus aureus*; MRCNS: methicillin-resistant coagulase-negative *Staphylococcus*; MRSA: methicillin-resistant *Staphylococcus aureus*.

**Table 5:** Comparative analysis of the clinical results (SF-36) of the Masquelet reconstruction of the upper and lower extremities.

Variable	Upper Extremity (*n* = 20)	Lower Extremity (*n* = 25)	*p*-Value
SF-36 functional (mean)			
Preoperation	81.6 (±8.11)	82.69 (±11.65)	0.779
Postoperation	34.6 (±26.6)	21 (±15.5)	0.107
SF-36 mental (mean)			
Preoperation	51.6 (±6.2)	48.84 (±9.46)	0.371
Postoperation	20.06 (±16.4)	13 (±5.61)	0.135
SF-36 total (mean)			
Preoperation	133.26 (±11.97)	131.53 (±20.44)	0.791
Postoperation	54.66 (±42.58)	34 (±19.37)	0.106

SF-36 Short Form Health Survey; Wilcoxon signed-rank test, *p*-value < 0.05.

## Data Availability

All data generated or analyzed during this study are included in the published article.

## Abbreviations

PMMA	Polymethylmethacrylate bone cement
LCP	Low-contact locking plate
IMN	Intramedullary nail
LLFI	Lower Limb Functional Index
DASH	Disabilities of the Arm Shoulder and Hand
IMT	Induced membrane technique
RIA	Reaming irrigation aspiration
AP	Antero-posterior
CT	Computed tomography
CRP	C-reactive protein
MRI	Magnetic resonance imaging
mm	Millimeters
cm	Centimeters
